# 

**Published:** 1916-02-05

**Authors:** 


### PASSING EVENTS AND TOPICS.

### A Cavell Street for London.

At the earliest opportunity London will have a thoroughfare named in commemoration of Nurse Cavell. The suggestion for this was made at a meeting of the L.C.C., and a promise was instantly given that it would be adopted. Already there are new Cavell thoroughfares on the Continent.

### Woman Doctor and a Corsican Hospital.

The Manchester and District Federation Field Hospital Committee is supporting a hospital at Ajaccio, in Corsica, for sick and wounded Serbian refugees, to which a military wing is to be added later. Dr. Mary Blair, with a party of nurses, has gone from Salonika to Corsica, where she has opened the hospital with initial accommodation for forty beds. The upkeep of the hospital is put at £400 a month. Dr. Blair has been asked by the authorities in Corsica to be the chief medical officer for all the Serbians in the island.

### Sir Clements Markham and Quinine.

The tragic death of Sir Clements Markham recalls the fact that it was to this great explorer that the task was entrusted of superintending the necessary arrangements for the collection of the cinchona plants in South America and their introduction into India. It is hardly correct, however, to attribute, as some of the obituary notices do, the reduction in the price of quinine "from many shillings to a few pence per ounce" to the cultivation of cinchona bark in India. This was mainly due to the Dutch Government, which succeeded in cultivating in Java a cinchona tree whose bark yielded a vastly higher percentage of quinine than did the Peruvian tree.

### Antiseptics at Sea.

An electrical apparatus has been installed in the *Aquitania*, under the supervision of Dr. Dakin and at the instance of the Medical Research Committee of the National Insurance Act, for the purpose of the production of sodium hypochlorite for use as an antiseptic for wounds and for disinfecting purposes. According to the *Times*, the cost of 100 gallons of a solution containing two parts of sodium hypochlorite to 1,000 pints works out at 3d. It is estimated that the economy in largely replacing expensive coal-tar disinfectants by electrolytic hypochlorite will pay for the cost of the apparatus in the course of a single trip of three weeks.

### Lectures for First-Aid Students.

A course of lectures on the anatomy of the body for first-aid and ambulance students will be given in the theatre of the Royal College of Surgeons, Lincoln's Inn Fields, by Professor Arthur Keith, Conservator of the Museum, at 5.30 p.m. on the under-mentioned dates :

February : Monday, 14; Wednesday, 16; Friday, 18; Monday, 21; Wednesday, 23; Friday, 25.

March : Monday, 13; Wednesday, 15; Friday, 17; Monday, 20; Wednesday, 22; Friday, 24; Monday, 27; Wednesday, 29; Friday, 31.

April : Monday, 3; Wednesday, 5; Friday, 7; Monday, 10; Wednesday, 12; Friday, 14; Wednesday, 26; Friday, 28.

May : Wednesday, 3; Friday, 5.

The course is open to all members of ambulance companies and of first-aid classes.

### The Book Market.

LIST OF NEW BOOKS RECEIVED FROM THE FOLLOWING PUBLISHERS:—

A. and C. Black: "The Englishwoman's Year Book, 1916." 2s. 6d. net. "Who's Who Year Book, 1916." 1s. net.

T. Fisher Unwin, Ltd.: "In a French Hospital." By M. Eydoux-Démians. 2s. 6d. net.

J. Whitaker and Sons, Ltd.: Whitaker's Almanack, 1916. 2s. 6d. net.

### Gifts and Bequests.

MR. J. WATSON HUGHES, of Mold, has given £500 for the purpose of extending the accommodation at Mold Cottage Hospital.

MISS REBECCA THORNE, of Elsham Road, Kensington, W., left about £12,000 in equal shares to the Central London Ophthalmic Hospital, the West London Hospital, and St. Mary's Hospital, Paddington.

THE Trustees of the Bath Municipal Charities have received a legacy of £1,000, less duty, under the will of Miss Mary Ann Wood, late of 26 Paragon, Bath, who died in May 1899. She left a life interest to Mr. James Wood, who died in July last. The actual amount received was £867 6s.

### Obituary.

WE regret to announce the death, on January 31, of Mr. A. W. Bodger, who for over twenty years has been secretary to the London Temperance Hospital. Mr. Bodger was fifty-two years of age, and had been in failing health for some time past, and his death came rather suddenly. Before his appointment to the secretaryship of the London Temperance Hospital, he was one of the joint secretaries to the Native Races Committee in connection with Liquor Traffic.

DR. HERBERT WILLIAMS, Medical Officer of Health for the Port of London, whose death occurred recently, received his medical training at St. Bartholomew's Hospital, where he subsequently held resident appointments. He entered the service of the Corporation of the City of London in 1892 as Assistant Port Medical Officer, succeeding to the chief officership nine years later, on the transfer of Dr. Collingridge to the medical officership of the City of London. One of the matters to which Dr. Williams gave special attention was that of improving the quarters of the crews on board ship.

Rates of Subscription: Twelve months, 6/6; Six months, 4/-; Three months, 2/-, post free to any address in the United Kingdom. Abroad, Twelve months, 8/8; Six months, 5/-; Three months, 2/6, post free.—Address The Subscription Dept., "The Hospital," The Hospital Building, 28/29 Southampton Street, Strand, London, W.C.

